# Identifying Optimal Candidates for Trimodality Therapy among Nonmetastatic Muscle-Invasive Bladder Cancer Patients

**DOI:** 10.3390/curroncol30120740

**Published:** 2023-11-29

**Authors:** Shengming Ran, Jingtian Yang, Jintao Hu, Liekui Fang, Wang He

**Affiliations:** 1 Department of Urology, Sun Yat-sen Memorial Hospital, Sun Yat-sen University, Guangzhou 510289, China; ranshm@mail2.sysu.edu.cn (S.R.); hujt7@mail2.sysu.edu.cn (J.H.); 2 Guangdong Provincial Key Laboratory of Malignant Tumor Epigenetics and Gene Regulation, Sun Yat-sen Memorial Hospital, Sun Yat-sen University, Guangzhou 510289, China; 3 Guangdong Clinical Research Center for Urological Diseases, Guangzhou 510289, China; 4 Department of Urology, The Third People’s Hospital of Shenzhen, Southern University of Science and Technology, Shenzhen 518116, China; yangjingtian97@foxmail.com

**Keywords:** nonmetastatic muscle-invasive bladder cancer, trimodality therapy, predictive model, SEER database, nomogram

## Abstract

(1) Background: This research aims to identify candidates for trimodality therapy (TMT) or radical cystectomy (RC) by using a predictive model. (2) Methods: Patients with nonmetastatic muscle-invasive bladder cancer (MIBC) in the Surveillance, Epidemiology, and End Results (SEER) database were enrolled. The clinical data of 2174 eligible patients were extracted and separated into RC and TMT groups. To control for confounding bias, propensity score matching (PSM) was carried out. A nomogram was established via multivariable logistic regression. The area under the receiver operating characteristic curve (AUC) and calibration curves were used to assess the nomogram’s prediction capacity. Decision curve analysis (DCA) was carried out to determine the nomogram’s clinical applicability. (3) Results: After being processed with PSM, the OS of the RC group was significantly longer compared with the TMT group (*p* < 0.001). This remarkable capacity for discrimination was exhibited in the training (AUC: 0.717) and validation (AUC: 0.774) sets. The calibration curves suggested acceptable uniformity. Excellent clinical utility was shown in the DCA curve. The RC and RC-Beneficial group survived significantly longer than the RC and TMT-Beneficial group (*p* < 0.001) or the TMT group (*p* < 0.001). However, no significant difference was found between the RC and TMT-Beneficial group and the TMT group (*p* = 0.321). (4) Conclusions: A predictive model with excellent discrimination and clinical application value was established to identify the optimal patients for TMT among nonmetastatic MIBC patients.

## 1. Introduction

Bladder cancer is the tenth most common cancer worldwide, with an estimated 549,000 new cases and 200,000 deaths in 2018 [1]. Incidence rates were higher in men for whom it was the sixth most common cancer. The highest incidence rates were seen in the developed world [1]. The highest incidence rates in Europe were observed in Southern Europe, e.g., Greece (5800 new cases and 1537 deaths in 2018), Spain, and Italy, and Western Europe, e.g., Belgium and the Netherlands. Tobacco smoking is the highest risk factor for developing bladder cancer, accounting for 50% of cases, followed by occupational exposure to aromatic amines and ionizing radiation [2]. Bladder cancer is classified as either non-muscle-invasive or muscle-invasive. Although muscle-invasive bladder cancer accounts for only around 30% of newly diagnosed cases, its aggressive nature, proclivity for metastasis, medication resistance, and high recurrence rate all contribute to a poor prognosis [3,4]. Up to 10% of patients have clinically evident metastases at diagnosis, and roughly one-third of individuals with localized MIBC will develop metastases after initial tumor therapy [5]. Without treatment, 41% of MIBC patients will die in six months, and the five-year overall survival rate is only 5% [6].

The two main treatments for localized bladder cancer are radical cystectomy and a bladder-sparing approach with trimodality therapy (TMT), which are both now endorsed in major guidelines [7,8]. TMT combines the maximal transurethral resection of the bladder tumor (TURBT) with concomitant concurrent radiation therapy (RT) and chemotherapy. The purpose of TMT is to preserve the bladder and quality of life without compromising the oncological prognosis.

With the administration of neoadjuvant or adjuvant chemotherapy, RC is still the conventional treatment for MIBC. However, given the strong effects of RC on the patient’s physical condition and complications after RC, such as urinary incontinence and loss of sexual function, there is a decline in the quality of life for these patients. Therefore, RC is not applicable to all patients with nonmetastatic MIBC. Alternative treatment options to RC are urgently needed for patients with muscle-invasive bladder cancer (MIBC) who are unable or unwilling to undergo radical cystectomy. For well-selected patients, TMT may be a reasonable alternative to radical cystectomy (RC) [9,10]. No completed RCTs have compared the oncologic outcome of TMT with RC, but TMT has been shown to provide an equivalent oncologic outcome in comparison to RC in terms of overall survival (OS) and disease-free survival (DFS) in some previous studies [11,12,13]. However, not all patients can achieve oncologic outcomes comparable to RC through TMT. The general acceptance of TMT needs more studies that address concerns about the recurrence of residual tumors. When compared with palliative care, trimodal therapy appears to be a curative alternative for older patients who are unable to undergo extensive surgery, and it may lead to better survival in these patients [14]. The inappropriate selection of TMT may result in adverse disease progression in patients. To avoid this, optimal bladder cancer patients for TMT should be identified through a reliable tool.

We developed a predictive model based on a public nationwide dataset to recognize the optimal MIBC patients for TMT, especially for whom radical cystectomy is not an option or acceptable.

## 2. Materials and Methods

### 2.1. Data Source and Patient Selection

The clinical data of bladder cancer patients were extracted from the Surveillance, Epidemiology, and End Results (SEER) database, including age, sex, race, primary site, histologic type, grade, TNM stage, etc. The N stage was determined with a combination of imaging techniques and through pathological findings obtained after lymph node dissection. For patients who did not undergo RC and for whom pathological N-staging was not available, the imaging-suggestive N stage was used. We were granted access to these clinical data from this publicly accessible database, and the research was performed in line with the Helsinki Declaration.

Cases of bladder cancers diagnosed from 2004 to 2017 were extracted. The inclusion criteria were (1) patients with nonmetastatic muscle-invasive bladder cancer (T2-4, N0/N+, M0) and (2) patients who received TMT or RC with neoadjuvant chemotherapy. The exclusion criteria were (1) patients with incomplete clinical data, including TMN stage, survival outcomes, treatment, pathological grade, and tumor size, and (2) patients with more than one primary tumor.

### 2.2. Statistical Analysis

According to the primary treatment, patients were separated into the TMT and RC groups. Patients who received TMT, including TURBT followed by RT and chemotherapy, were assigned to the TMT group. Another group of patients underwent neoadjuvant chemotherapy and RC. We used propensity score matching (PSM) to mitigate the impact of confounding bias in asymmetric variables in nonrandomized studies. All the variables were included in the propensity score model. PSM was carried out with no replacement using the nearest-neighbor method with a caliper width of 0.05 at a ratio of 1:1. Before and after PSM, the t-test, the Kruskal–Wallis test, and Fisher’s exact test were used to determine the statistically significant differences in continuous and categorical variables. The Kaplan–Meier survival method was used to estimate OS, which was then compared with the log-rank test.

### 2.3. Construction, Validation, and Clinical Application of the Nomogram

We supposed that patients who received RC and survived beyond the median OS of the TMT group in the matched cohort could benefit from RC and vice versa from TMT. Based on this assumption, patients in the RC group were separated into the TMT-Beneficial group (OS ≤ 29 months) and the RC-Beneficial group (OS > 29 months). In the RC group, living patients who were followed up for less than 29 months at the time of the last recording were excluded from the predictive model. Logistic regression was performed to ascertain the independent variables, which were then included in the nomogram.

In the RC group, the training and validation sets were randomly distributed in a ratio of 7:3. Then, to predict TMT- or RC-beneficial patients, a multivariable logistic regression model was established and displayed in a nomogram. The logistic regression model comprised the predictor variables, including age, histologic type, T, and N. On the basis of multivariate analysis in the training set, a nomogram was provided to offer a quantitative tool for predicting optimal MIBC patients for RC or TMT. After summing the scores for each variable, the probability that nonmetastatic MIBC patients would benefit from RC or TMT was determined.

The area under the receiver operating characteristic (ROC) curve and calibration plots were performed to assess the nomograms’ discriminative capacity and accuracy. In addition, the distinguishability of the model was validated in all nonmetastatic MIBC patients after PSM. According to the probability of benefit, patients in the RC group were separated into an RC and RC-Beneficial group with over 50% beneficial probability and an RC and TMT-Beneficial group with less than 50% probability of benefit. We performed Kaplan–Meier analysis and the log-rank test for comparison with the OS among the RC and RC-Beneficial group, the RC and TMT-Beneficial group, and the TMT group, which tested the discriminative ability. Decision curve analysis (DCA) was conducted to determine the nomogram’s applicability.

Statistical analysis was carried out with SPSS (version 26.0) (IBM Corp, Armonk, USA) and R (version-4.0.5; http://www.Rproject.org accessed on 1 October 2023). A *p*-value < 0.05 was considered statistically significant.

## 3. Results

### 3.1. Selection of Patients and Characteristics

**Figure 1:**
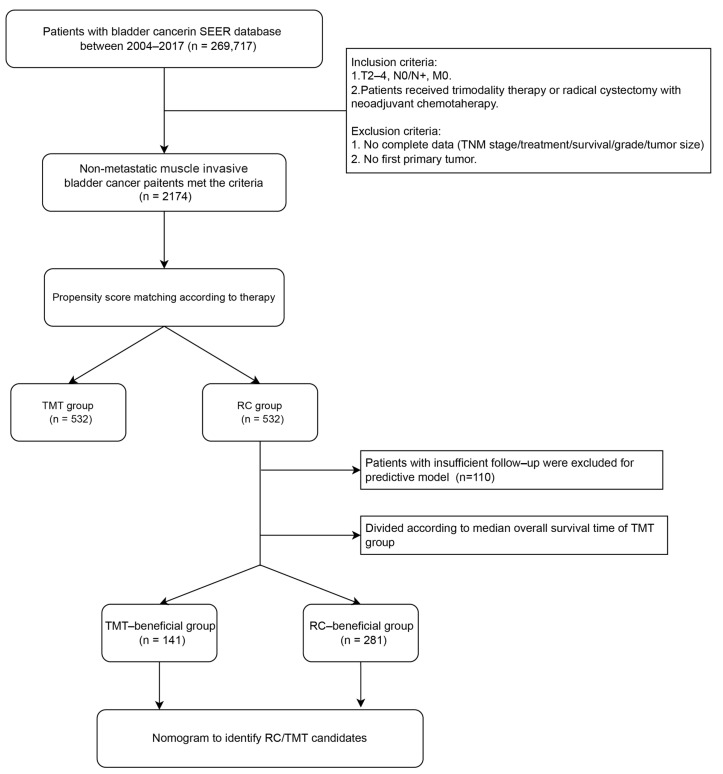
Flowchart of the study. SEER: Surveillance, Epidemiology, and End Results; TMT: trimodality therapy; RC: radical cystectomy.

The clinical data of 269,717 bladder cancer patients were extracted from the SEER database from 2004 to 2017. According to the criteria, 2174 eligible nonmetastatic MIBC patients were enrolled (Figure 1). Before PSM, 1170 patients (53.8%) received RC treatment. Imbalanced baseline characteristics were observed in age, sex, primary site, T stage, and N stage between the two groups (Table 1). After PSM included all variables at a 1:1 ratio, 532 matched pairs of nonmetastatic MIBC patients receiving RC or TMT were enrolled in the subsequent analysis. Each variable was considerably balanced after PSM (all *p* > 0.2), which is shown in Table 1.

### 3.2. Survival in Nonmetastatic MIBC Patients

**Figure 2:**
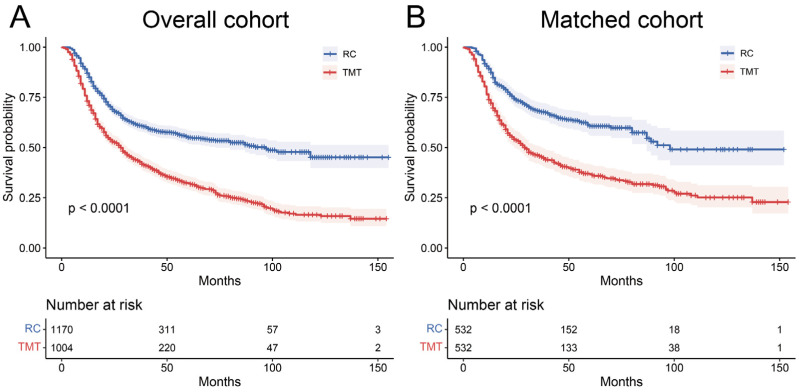
Kaplan–Meier curves of overall survival in nonmetastatic muscle-invasive bladder cancer patients according to treatment before (**A**) and after (**B**) propensity score matching. TMT, trimodality therapy; RC, radical cystectomy.

As shown in Kaplan–Meier curves (Figure 2), significant differences in OS were demonstrated between the RC and TMT groups in the overall and matched cohorts (both *p* < 0.001). The matched cohort was enrolled in the following analysis. Patients who underwent RC survived for a longer median OS (43 vs. 29 months; *p* < 0.001).

### 3.3. Nomogram to Identify Candidates for RC or TMT

In the RC group, 110 living patients, who were followed up for less than 29 months at the time of the last recording, were excluded from the predictive model because the beneficial treatment could not be determined. On the basis of the median OS (29 months) in the TMT group, 281 patients (OS > 29 months) were assigned to the RC-Beneficial group, and 141 patients who survived shorter than 29 months could not benefit from RC (TMT-Beneficial group).

**Figure 3:**
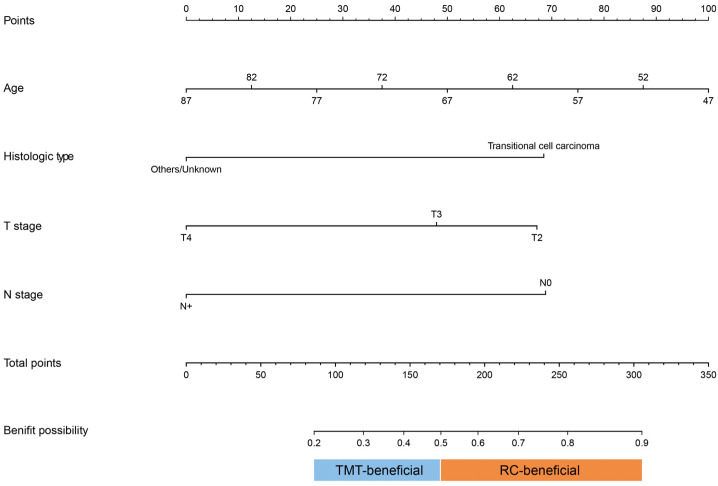
A nomogram to predict optimal candidates for TMT or RC. TMT, trimodality therapy; RC, radical cystectomy.

According to the results of the logistic regression (Table 2), independent factors that affect the beneficial probability for treatment were enrolled in the nomogram, including age, histologic type, T stage, and N stage. A nomogram to determine the ideal candidates for RC or TMT was set up (Figure 3).

### 3.4. Validation and Clinical Application of Prediction Nomogram

**Figure 4:**
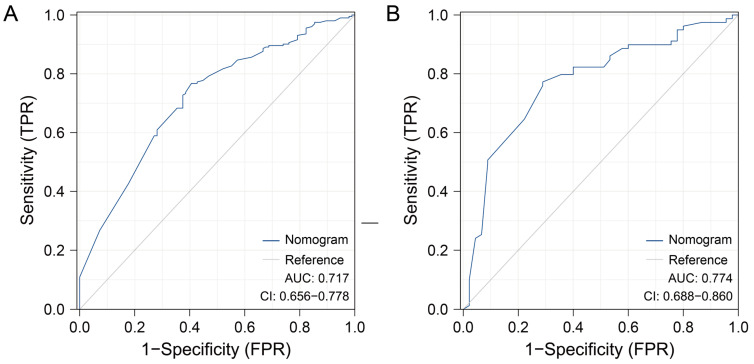
Receiver operating characteristic curve of the nomogram in the training (**A**) and validation (**B**) sets. TPR, true positive rate; FPR, false positive rate; AUC, area under curve; CI, confidence interval.

**Figure 5:**
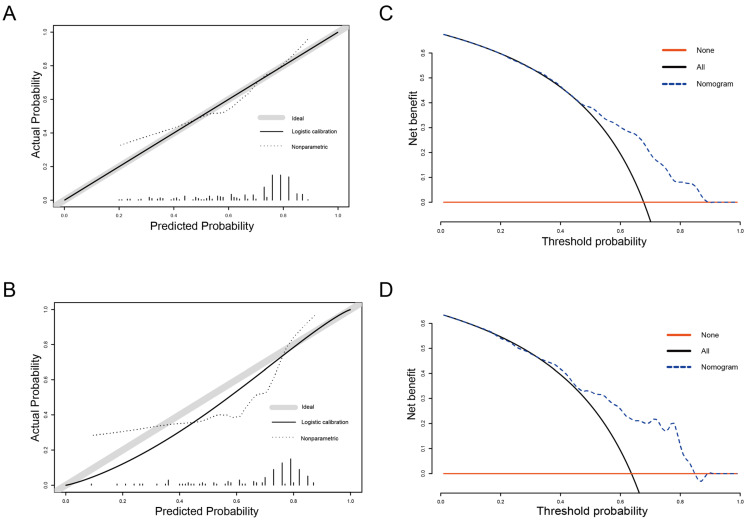
Calibration curves of the nomogram in the training (**A**) and validation (**B**) sets. Decision curve analysis for the prediction nomogram in the training (**C**) and validation (**D**) sets.

**Figure 6:**
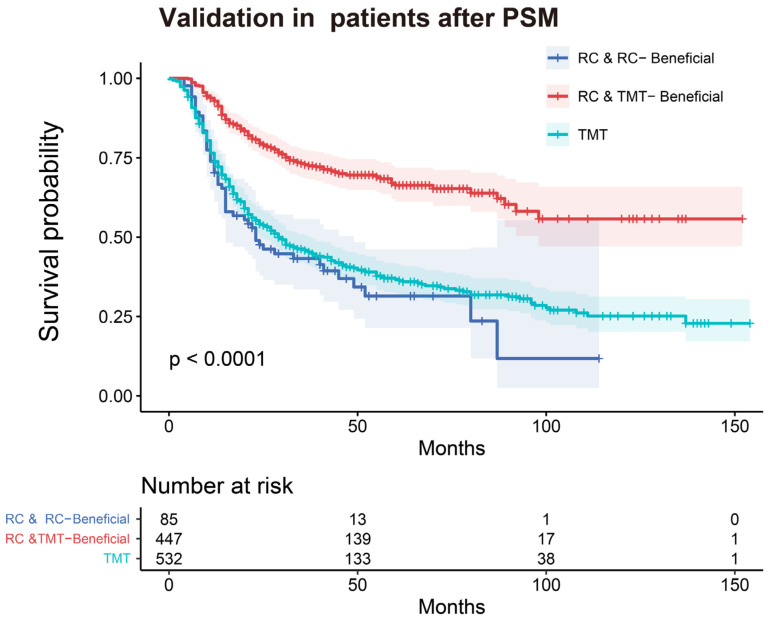
Kaplan–Meier curves of overall survival in the RC and RC-Beneficial group, the RC and TMT-Beneficial group, and the TMT group. PSM, propensity-score-matching; TMT, trimodality therapy; RC, radical cystectomy.

As shown in Figure 4, excellent discrimination capacity was shown in the training and validation sets (AUC = 0.717 and 0.774, respectively). In addition, a good correlation was demonstrated in the calibration plots of the nomogram between the actual observation and prediction by nomogram (Figure 5). The discriminative capacity of the model was further verified via Kaplan–Meier analysis and the log-rank test in the nonmetastatic MIBC patients after PSM (Figure 6). The RC and RC-Beneficial group survived significantly longer than the RC and TMT-Beneficial group (*p* < 0.001) and the TMT group (*p* < 0.001), but no significant difference was found between the RC and TMT-Beneficial group and the TMT group (*p* = 0.321).

Curves for DCA were calculated to assess the clinical value of the application (Figure 5). For nonmetastatic MIBC patients, DCA revealed that utilizing the nomogram to predict treatment benefit probability offered a better net benefit than either the “treat all with RC” or “treat all with TMT” methods, demonstrating the applicability of our model. By applying our predictive model, patients who would otherwise be ineligible for radical surgery can avoid surgical trauma, thereby improving the quality of survival.

## 4. Discussion

RC is still a gold-standard treatment for nonmetastatic MIBC. Despite this, not all nonmetastatic MIBC patients are suitable for RC and could gain an improved survival outcome. Some studies have confirmed that a bladder-sparing TMT treatment should be a treatment option available to all patients with cN+ M0 bladder cancer [15]. For these patients, being treated with TMT has favorable oncological outcomes similar to being treated with RC [16]. TMT may be the ideal treatment solution to bring about an improved quality of life without decreased tumor control. Although TMT has now been included in the current guidelines as a possible treatment option for MIBC, it is still unclear which patients would benefit from TMT rather than RC. Aiming to identify and classify optimal candidates for TMT and describe their clinical characteristics, we built a nomogram incorporating age, histologic type, T stage, and N stage.

Although a randomized controlled trial comparing RC and TMT is lacking at present, the use of radiation therapy in bladder preservation has been championed by the Radiation Therapy Oncology Group (RTOG) for an extended period. Prospective data from the RTOG have shown comparable long-term clinical results to cystectomy series, especially when considering modern treatment approaches [17]. Recent results have shown that TMT could offer considerable long-term survival rates compared with RC [11,12,13,18,19]. Another study demonstrated high rates of complete response and bladder preservation in patients receiving TMT and confirmed disease-specific survival rates similar to modern cystectomy series [20]. Even in terms of long-term outcomes, the 10-year bladder preservation rate in an experienced medical center can reach 79%, and the 10-year OS and CSS rates can reach 43.2% and 76.3%, respectively [21]. The Massachusetts General Hospital (MGH) experience also showed that 72% of patients with cT2-4a disease had a complete response after concurrent cisplatin-based chemotherapy and RT after TURBT, and only 22% of them needed cystectomy.

We confirmed the clinical and pathological predictors of overall survival after TMT or RC, including age, histologic type, T stage, N stage, and so on. In the model, age was the strongest predictor of patients who would benefit from TMT. In this study, older patients were more likely to benefit from TMT. This result may be explained by the fact that older patients tend to be unable to tolerate RC. Meanwhile, the high rate of perioperative complications after cystectomy is partly due to the age of the patient [22]. Consistent with previous studies, age at diagnosis, pathological type, and clinical stage were correlated with local recurrence and poor survival outcomes [23,24,25,26,27]. For lymph-node-negative MIBC, TMT without neoadjuvant chemotherapy or dose intensification was still reported to be related to a high complete response rate and bladder-preserving rate [25]. The earlier T stage and N stage may be related to the reduced surgical difficulty and associated risks of perioperative complication, which are associated with better outcomes. A retrospective study of 303 patients showed that variant histology did not affect the survival outcomes of TMT, which compared outcomes of pure urothelial carcinoma with variant urothelial carcinoma after TMT [26]. However, our results demonstrated that urothelial carcinoma has a greater possibility of benefit from RC rather than TMT, compared with non-urothelial carcinoma. Non-urothelial variant histology, including squamous cell carcinoma and adenocarcinoma, appears to have a more aggressive natural history and worse survival outcomes [28,29]. In our results, good differentiation and moderate differentiation (G1/2) accounted for a very low proportion, and the pathological grade was not the independent factor in the logistic regression. Hence, the pathological grade was not included in the predictive model. Furthermore, the response of non-urothelial bladder cancer to chemoradiation is largely unknown. Different from other studies, our results showed patients who underwent TMT did not have prolonged survival outcomes. The TURBT technique, the completeness of resection, and the regimens of chemoradiation all can affect the outcome of TMT. Generally speaking, not all patients can benefit from TMT. In order to obtain the best possible oncological results, it is imperative to conduct a comprehensive evaluation of patients prior to commencing TMT. Therefore, the selection of patients who can obtain comparable survival outcomes from TMT is of utmost importance. There are no definite selection criteria for optimal TMT patients. Ideal candidates for TMT in a previous study included a solitary cT2 tumor without extensive carcinoma in situ, macroscopically complete TURBT, the absence of hydronephrosis, and so on [30]. Some scholars believe that the assessment of complete histological responses with TURB after NAC may help in selecting the best candidates for bladder preservation with TMT [16].

Thus, this exploratory study has contributed to identifying the optimal candidates for TMT by developing an individualized prediction model. Through the nomogram, patients who suit TMT were individually identified via the prediction of their benefit potential, which was of considerable value in assessing the treatment efficacy. The nomogram we established was an applicable implementation to identify suitable nonmetastatic MIBC patients for TMT or RC. As shown in Figure 6, through the application of our predictive model, patients who would otherwise be ineligible for radical surgery, which cannot provide improved survival, can avoid surgical trauma, probably resulting in greater quality of life. In addition, rational treatment regimens can provide MIBC patients with longer survival times and reduce treatment costs.

TMT is a bladder-sparing treatment strategy that has several advantages when compared with radical cystectomy (RC). Firstly, in terms of preserving the native bladder of patients with MIBC, TMT offers a better general quality of life compared with RC. TMT offers better physical, role, social, emotional, and cognitive functioning; better bowel function; fewer bowel symptoms; better sexual function; and a better body image compared with RC [31]. Regarding urinary function, the MGH experience also demonstrated that, after TMT, the majority of conserved bladders functioned normally at a median follow-up of 6 years [32]. Another study focusing on female patients who received TMT additionally reported a high OS and the excellent functional quality of the conserved bladder, the rectum, and the vagina [33]. Royce et al. carried out a Markov model to compare quality-adjusted life years (QALYs) between TMT and RC in MIBC patients and found that TMT was more efficient with an incremental gain of 1.61 QALYs [34]. The results further confirm that TMT is still an essential option for MIBC patients desiring a better quality of life. Secondly, TMT has a low level of toxicity. No patients required cystectomy because of treatment-related toxicity after TMT using Massachusetts General Hospital (MGH) protocols [20]. Thirdly, TMT has fewer operational obstacles. RC is a complicated surgical technique that represents major difficulties for hospitals and physicians [35]. In comparison, TMT is relatively easier to apply and can be carried out in most general hospitals. TMT, on the other hand, necessitates more patient compliance and has higher long-term expenditures [36].

Chemotherapy and radiotherapy are the essential parts of TMT. Nearly half of MIBC patients who receive RC still have tumor recurrences [37], which shows that even RC alone cannot achieve the desired tumor control. For bladder preservation treatment, patients who respond to neoadjuvant chemotherapy are more likely to achieve bladder preservation, and this has become a significant predictive factor for both overall survival and disease-specific survival [20,27]. A study [38] including 104 nonmetastatic MIBC patients who underwent three cycles of neoadjuvant chemotherapy showed that nearly half of the patients were T0 after receiving neoadjuvant methotrexate, vinblastine, doxorubicin, and cisplatin (M-VAC) chemotherapy. Furthermore, 44% of patients receiving neoadjuvant M-AVC chemotherapy with or without TURBT maintained an intact bladder. The results of a long-term follow-up also demonstrated that TURBT and a complete response after induction therapy are still independent correlates of increased survival outcomes [21].

Improvements in the TURBT technique and radiation therapy have been limited in recent years. The discovery of cancer immune surveillance and immunotherapy has improved the treatment options for MIBC patients undergoing TMT. Although the combination of immunotherapy and radiotherapy is in the early stages of clinical testing, as the efficacy of immunotherapy is being confirmed by relevant clinical data, the exploration of immunotherapy for bladder cancer indications has gradually moved from second-line to first-line treatment. Immunotherapy has been applied in the second-line treatment of unresectable or metastatic bladder cancer and the first-line treatment of PD-L1-positive patients unsuited for platinum-based chemotherapy. Several studies have provided preliminary evidence suggesting that patients who attain a pathological full response following neoadjuvant therapies in combination with the maximum transurethral resection of the bladder tumor may be suitable candidates for bladder preservation therapy [39]. With the increasing application of immunotherapy in bladder preservation therapy, exploratory studies to help identify the optimal candidates for TMT are urgent and necessary. Optimal patients for TMT should be precisely identified before receiving individual treatments including chemoradiotherapy and immunotherapy. Our predictive model fills the gap in accurately predicting MIBC patients who can benefit from TMT rather than RC. This could boost the development of TMT and the comprehensive treatment of bladder cancer in the future.

There are some limitations to our study. First of all, inherent biases are inevitable in an observational study, such as the effect of confounding factors. We performed propensity score matching to eliminate the effects of confounders. Furthermore, we eliminated patients whose information was unknown, which was another important source of bias in the selection process. Secondly, the SEER database has certain restrictions. For example, the SEER database gathers vast amounts of patient data from many locations and institutions, and it was difficult to reconcile the disparities in treatment and pathological assessment criteria. Furthermore, the SEER database lacked key essential information for nonmetastatic MIBC patients, including as chemotherapy medication, perioperative mortality statistics, and radiation treatment duration. Concurrently, another limitation of this study is the lack of information on immunotherapy and target therapy, which provides more treatment options for TMT [40]. Also, we did not include endpoint indicators such as cancer-specific mortality. Lastly, even though the validation cohort underwent internal verification, the accuracy of this verification technique was compromised because the patients in both the training and validation sets were drawn from the same database. For external validation, a sizable prospective clinical trial is necessary.

In conclusion, we screened nonmetastatic MIBC patients who would benefit from TMT or RC. There is no additional cost associated with this prediction model. The prediction model deserves additional prospective validation, and it is necessary to enhance the predictive model after the incorporation of more variables in the future. Our prediction model, once validated in a prospective cohort, could assist clinical decision making and be remarkably valuable. It is posited that a promising avenue for future study in this domain entails the integration of immunotherapy and targeted therapeutics in conjunction with established modalities.

## Figures and Tables

**Table 1:** Baseline characteristics of the study population.

Variable	Overall Cohort		*p*	Matched Cohort		*p*
	RC	TMT		RC	TMT	
	*n* = 1170(%)	*n* = 1004(%)		*n* = 532(%)	*n* = 532(%)	
Age (median (IQR))	62.00 [57.00, 72.00]	77.00 [67.00, 82.00]	<0.001	67.00 [62.00, 72.00]	67.00 [62.00, 72.00]	0.999
Gender			0.001			1.000
Male	782 (66.8)	738 (73.5)		386 (72.6)	385 (72.4)	
Female	388 (33.2)	266 (26.5)		146 (27.4)	147 (27.6)	
Race			0.752			0.272
White	1017 (86.9)	868 (86.5)		470 (88.3)	457 (85.9)	
Others/unknown	153 (13.1)	136 (13.5)		62 (11.7)	75 (14.1)	
Primary site			0.020			0.999
Trigone of bladder	83 (7.1)	74 (7.4)		40 (7.5)	39 (7.3)	
Bladder neck	24 (2.1)	28 (2.8)		11 (2.1)	11 (2.1)	
Lateral wall of bladder	255 (21.8)	253 (25.2)		122 (22.9)	119 (22.4)	
Posterior wall of bladder	91 (7.8)	102 (10.2)		49 (9.2)	51 (9.6)	
Others/Unknown	717 (61.3)	547 (54.5)		310 (58.3)	312 (58.6)	
Histologic type			1.000			0.845
Urothelium carcinoma	1058 (90.4)	908 (90.4)		475 (89.3)	472 (88.7)	
Others/unknown	112 (9.6)	96 (9.6)		57 (10.7)	60 (11.3)	
Grade			0.090			0.862
G 1/2	31 (2.6)	40 (4.0)		18 (3.4)	16 (3.0)	
G 3/4	1139 (97.4)	964 (96.0)		514 (96.6)	516 (97.0)	
T stage			<0.001			0.772
T 2	590 (50.4)	837 (83.4)		379 (71.2)	381 (71.6)	
T 3	391 (33.4)	91 (9.1)		81 (15.2)	86 (16.2)	
T 4	189 (16.2)	76 (7.6)		72 (13.5)	65 (12.2)	
N stage			<0.001			0.428
N 0	325 (27.8)	76 (7.6)		80 (15.0)	70 (13.2)	
N +	845 (72.2)	928 (92.4)		452 (85.0)	462 (86.8)	
Tumor size, mm (mean (SD))	45.16 (34.83)	45.99 (20.76)	0.511	46.29 (44.95)	46.45 (20.83)	0.940

PSM: propensity score matching; TMT: trimodality therapy; RC: radical cystectomy.

**Table 2:** Logistic regression to determine independent factors for beneficial probability.

Covariate	Regression Coefficient (SE)	Odds Ratio (95% CI)	*p*
Age	−0.035 (0.015)	0.966 (0.938–0.994)	0.017
Gender			
Male	References		
Female	−0.058 (0.262)	0.944 (0.565–1.578)	0.826
Race			
White	Reference		
Others/unknown	0.004 (0.365)	1.004 (0.491–2.054)	0.992
Primary site			
Trigone of bladder	Reference		
Bladder neck	0.428 (1.04)	1.534 (0.2–11.779)	0.68
Lateral wall of bladder	0.015 (0.478)	1.015 (0.397–2.591)	0.975
Posterior wall of bladder	0.401 (0.605)	1.494 (0.457–4.889)	0.507
Others/unknown	−0.263 (0.435)	0.769 (0.328–1.804)	0.546
Histologic type			
Urothelium carcinoma	Reference		
Others/unknown	−1.254 (0.394)	0.285 (0.132–0.618)	0.001
Grade			
G 1/2	Reference		
G 3/4	−0.9 (0.664)	0.407 (0.111–1.493)	0.175
T stage			
T 2	Reference		
T 3	−0.778 (0.3)	0.459 (0.255–0.826)	0.009
T 4	−1.277 (0.336)	0.279 (0.144–0.539)	<0.001
N stage			
N 0	Reference		
N +	−1.127 (0.325)	0.324 (0.171–0.613)	0.001
Tumor size	0.004 (0.004)	1.004 (0.997–1.012)	0.272

SE: standard error; CI: confidence interval.

## Data Availability

Publicly available datasets were analyzed in this study. These data can be found here: https://seer.Cancer.gov/ (accessed on 20 February 2023).
